# Application of 2D Non-Graphene Materials and 2D Oxide Nanostructures for Biosensing Technology

**DOI:** 10.3390/s16020223

**Published:** 2016-02-06

**Authors:** Kateryna Shavanova, Yulia Bakakina, Inna Burkova, Ivan Shtepliuk, Roman Viter, Arnolds Ubelis, Valerio Beni, Nickolaj Starodub, Rositsa Yakimova, Volodymyr Khranovskyy

**Affiliations:** 1 National University of Life and Environmental Sciences of Ukraine, Kiev 03041, Ukraine; shavanova@gmail.com (K.S.); inna.burkova@ukr.net (I.B.); nikstarodub@yahoo.com (N.S.); 2 Institute of Biophysics and Cell Engineering of National Academy of Sciences of Belarus, Minsk 220072, Belarus; bakakinay@mail.ru; 3 Department of Physics, Chemistry and Biology (IFM) Linköping University, Linköping 58183, Sweden; ivan.shtepliuk@liu.se (I.S.); roy@ifm.liu.se (R.Y.); 4 University of Latvia, 19, Raina blvd., Riga 1586, Latvia; viter_r@mail.ru (R.V.); arnolds@latnet.lv (A.U.); 5 Biosensors and Bioelectronics Centre, Linköping University, Linköping 58183, Sweden; valerio.beni@ifm.liu.se

**Keywords:** two-dimensional materials, beyond graphene, transition metal dichalcogenides, transition metal oxides, two-dimensional oxides, transducers, biosensors

## Abstract

The discovery of graphene and its unique properties has inspired researchers to try to invent other two-dimensional (2D) materials. After considerable research effort, a distinct “beyond graphene” domain has been established, comprising the library of non-graphene 2D materials. It is significant that some 2D non-graphene materials possess solid advantages over their predecessor, such as having a direct band gap, and therefore are highly promising for a number of applications. These applications are not limited to nano- and opto-electronics, but have a strong potential in biosensing technologies, as one example. However, since most of the 2D non-graphene materials have been newly discovered, most of the research efforts are concentrated on material synthesis and the investigation of the properties of the material. Applications of 2D non-graphene materials are still at the embryonic stage, and the integration of 2D non-graphene materials into devices is scarcely reported. However, in recent years, numerous reports have blossomed about 2D material-based biosensors, evidencing the growing potential of 2D non-graphene materials for biosensing applications. This review highlights the recent progress in research on the potential of using 2D non-graphene materials and similar oxide nanostructures for different types of biosensors (optical and electrochemical). A wide range of biological targets, such as glucose, dopamine, cortisol, DNA, IgG, bisphenol, ascorbic acid, cytochrome and estradiol, has been reported to be successfully detected by biosensors with transducers made of 2D non-graphene materials.

## 1. Introduction

The developments in material science are the driving force of technological progress. In addition, it may be strongly argued that the creation of new materials of different dimensionality and functionality is the primary prerequisite for any likely significant breakthroughs to be made. The invention of graphene has unambiguously demonstrated that the properties of two-dimensional (2D) materials can be different and in many ways far superior to those of the bulk counterpart.

Graphene, being one atom-thick carbon nanosheets, became the first 2D nanostructure, which was isolated from parent graphite in 2004 [1]. It has served as a model for a two-dimensional system that has captured the interest of researchers from different fields, such as electronics, photonics, material science, engineering and sensing. In particular, graphene derivatives have been actively studied in the field of electrochemistry because of their unique physical and chemical properties in comparison to other carbon materials, such as large specific surface area (2630 m^2^/g) [2], superior electrical conductivity (200 S/m) [3,4], excellent thermal stability with oxidation resistance temperatures up to 600 °C [5], remarkable mechanical strength with Young’s modulus of around 1.0 TPa [6], outstanding optical transmittance of 97.7% [7] and high thermal conductivity between 3080 and 5150 W/mK [8]. It also demonstrates fascinating electrochemical properties, including wide electrochemical potential, activity and low charge-transfer resistance [9,10,11].

However, graphene, being the most well-known 2D crystal with a plethora of unique properties, has its disadvantages, which limit its applications. For instance, the lack of an intrinsic band gap is one of the largest obstacles on its way to be fully utilized. Fortunately, graphene’s discovery has triggered enormous interest toward other 2D materials and 2D nanostructures with possibly even more superior properties.

**Figure 1:**
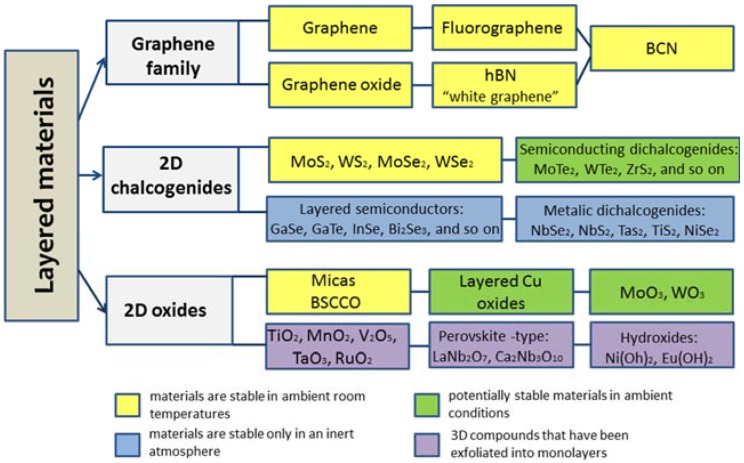
Chart illustrating the categorized library of 2D materials. Data are adapted from [16]. hBN is a hexagonal boron nitride; BCN is 2D nanocomposites containing boron, carbon and nitrogen; BSCCO is bismuth strontium calcium copper oxide.

With this in mind, the completely separate “beyond graphene” area of material science has been recently established and is growing extremely rapidly at present: following the success of graphene, the isolated monolayers and few-layer crystals of hexagonal boron nitride (hBN), transition metal dichalcogenides (TMDCs: MoS_2_, MoSe_2_, WS_2_, WSe_2_, *etc.*), transition metal oxides (TMOs: LaVO_3_, LaMnO_3_), transition metal chalcogenides (NbSe_3_, TaSe_3_) and others (Li_7_MnP_4_, MnP_4_), as well as layered complex oxides have been successfully fabricated [12]. More recently, the 2D analogues of the classical semiconductors, silicene and germanene have been studied [13], being most recently followed by the somewhat unexpected phosphorene [14]. The resulting pool of 2D crystals is therefore huge and covers a range of properties: from the most insulating to the best conductors, from the strongest to the softest. Recently, a number of excellent reviews were published [15], reflecting the growing library of the 2D materials. The summarized data are presented as a chart in Figure 1.

**Figure 2:**
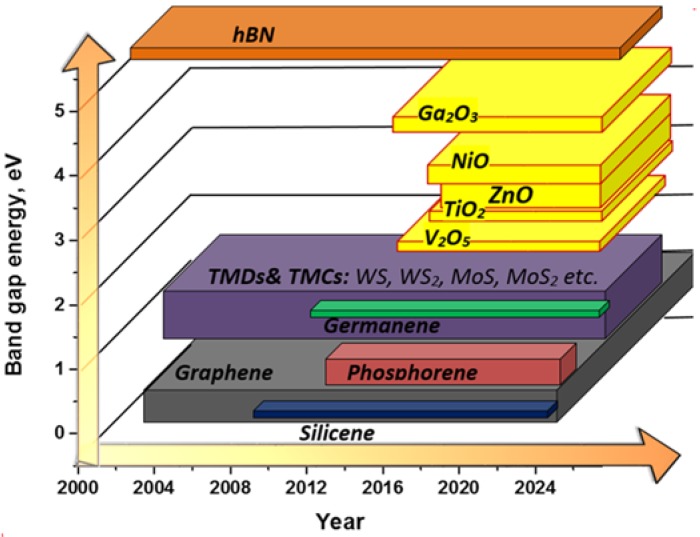
Illustration of the evolution of the family of 2D materials as a function of time (horizontal axis) and their respective band gap values (vertical axis). The yellow components represent the expected 2D metal oxides contribution.

However, in order to make rapid progress, what is ideally needed is a more complete library of 2D materials of matching semiconductor and electronic properties combined with new technologies for their fabrication on a commercial scale. The mentioned obstacle of the lack of band gap in graphene can be successfully overcome not only by existing 2D non-graphene materials, but also by a forthcoming group of metal oxides, which are able to fill the missing band gap energy range (~2.3–4.9 eV) (Figure 2).

However, to date, the technology for obtaining 2D materials has been inherently related to their layered structure and the weak van der Waals bonds that exist between the layers: the earliest scotch-tape approach was later transformed to the chemical intercalation and exfoliation of 2D flakes, and only recently has attention turned to the direct growth techniques (chemical vapor deposition (CVD) of Gr, TMDs and others); while metal oxides, e.g., TiO_2_, MnO_3_, WO_3_, mica and perovskite-like crystals, are only represented in the 2D materials family very modestly, highlighting once again the need for further detailed studies of their growth and properties. Herein and further in the article, we will call the group of 2D metal oxides and similar (e.g., one or a few atoms thin layers) the 2D metal oxide nanostructures.

In this article, we intentionally will not describe the methods and approaches for 2D non-graphene material synthesis, but recommend to our readers several comprehensive review articles, which were published recently and are devoted specifically to the technology of 2D materials fabrication (e.g., [13,17,18]).

Interest in the metal oxides in the 2D form is particularly strong in the context of biosensor applications. Among the various transducer materials that have been developed, nanostructured metal oxides have exceptional optical and electrical properties that offer excellent prospects for the interfacing of biological recognition events with electronic or optical signal transduction and for designing of a new generation of bioelectronics devices that may exhibit novel functions.

## 2. Principle of Biosensors Operation and Current Trends in Biosensing Technology

### 2.1. Biosensors Design and Principles of Operation

A biosensor is an analytical device that transforms a biological recognition event into another signal, e.g., optical, chemical, electrical or physical signal, that can be measured and quantified in real time [19].

**Figure 3:**
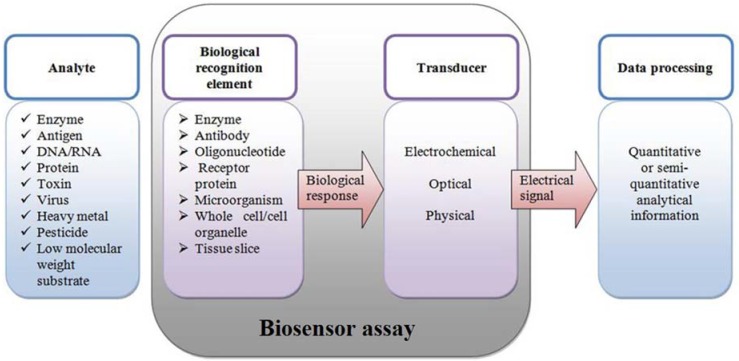
Principle of biosensor operation and main components: the analyte is detected by the biologically-affined sensitive layer, immobilized on the transducer. The biological response is transformed to an electrical, optical or electrochemical signal by the transducer and then further processed, providing the information. Data are summarized from [20,21].

Technologically, a biosensor is an integrated miniaturized device that has a biosensitive layer, connected to a transducing system for signal detection. The biosensitive layer is created by immobilization of the biological recognition element (enzyme, antibody, oligonucleotide, receptor protein, microorganism or the whole cell) on the surface of the biosensor (Figure 3). The biosensitive layer should be bioselective and sensitive to capture the appropriate analyte (enzyme, antigen, DNA/RNA, toxin, virus, heavy metal, pesticide, *etc.*) and interpret accurately the bio-recognition event. Biosensors integrate the selectivity of biomolecules and the processing power of modern microelectronics and optoelectronics [20].

**Figure 4:**
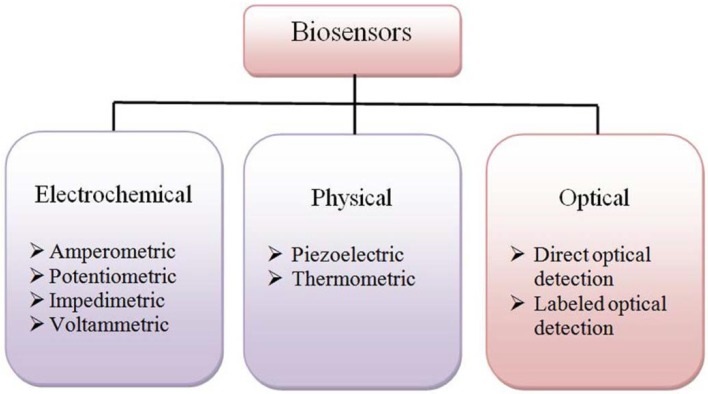
Types of biosensors based on their transducer operation.

Based on the detection method and transducer system, biosensors may be classified respectively into electrochemical, physical or optical (Figure 4).

Electrochemical biosensors are defined as integrated devices that provide specific quantitative or semi-quantitative analytical information using a biological recognition element, which is in contact with an electrochemical transduction element. Electrochemical biosensors are usually based on potentiometry and amperometry. The amperometric biosensors are the most successfully commercialized devices among numerous types of biosensors, since the research in the field of biosensors started with them [21]. Recent advances in electrochemical biosensors have been reviewed extensively by Lin *et al.* [22,23,24].

A physical transducer system includes piezoelectric and thermometric types of biosensors. Piezoelectric biosensors are based on an alternating potential and produce a standing wave in a crystal at a characteristic frequency. This frequency is highly sensitive to the surface properties of a crystal. If a crystal is coated with a biological recognition element, binding of a target analyte to a receptor will produce a change in the resonant frequency.

Thermometric biosensors are constructed by combining enzymes with temperature sensors. When the analyte is exposed to the enzyme, the heat of the enzymatic reaction is measured and calibrated against the analyte concentration [25].

Optical biosensors detect changes in the absorbance, photoluminescence (PL) or fluorescence of an appropriate indicator and changes in the refractive index [25]. The basic idea of optical biosensors is to produce an electronic signal, which is proportional in intensity or frequency to the concentration of a specific analyte or group of analytes, to which the biosensing element binds [26].

### 2.2. Current Trends in Biosensors

Since 1962, groups from all over the world have joined biosensor research from the moment when Clark and Lyons [27] designed the first amperometric biosensor by immobilizing of glucose oxidase on an oxygen electrode [28].

Today, due to recent advances, the definition of a biosensor has evolved from the classical concept of an enzyme-electrode to a variety of analytical methods based on biocatalysis and bioaffinity [29]. The improvement of biological components, the implementation of micro- and nano-technologies and the development of new methods of integration between bioreceptors and transducers promise rapid progress in biosensor technology [30]. As a result, biosensor research has become an interdisciplinary field that integrates state-of-the-art achievements in physics, biology, chemistry, material science, engineering, mathematics and information technologies [19]. In the past few decades, biosensors, which come in a large variety of sizes and shapes, have found applications, such as environmental and industrial monitoring, medicine, biotechnology, food analysis and production monitoring, healthcare, agriculture, as well as national security and defense [19,25]. Recently, the successful use of biosensors for environmental and industrial analysis, such as monitoring the microbiological and chemical quality of water [31,32], rapid detection of various toxins (bacterial, dinoflagellate toxins, mycotoxins, plant toxins) [32,33] and trace-level toxic heavy metal ions [34,35,36] and monitoring the concentration of different pesticides and their residues in food, water and soil [37], were reported. Biosensors are currently widely used in clinical diagnostics to determine the blood parameters (pH, pCO_2_ and pO_2_) [38], glucose, lactate, urea, creatinine, cholesterol and triglyceride monitoring [39,40,41], testing of genetic and infectious diseases [42], mutational analysis [43], skin allergy test [44] and cancer diagnostics [45]. Biosensors are the main resources to be utilized in the forthcoming era of point-of-care diagnostics. Diverse biosensing devices are considered to be applicable for point-of-care sensing systems. Recently, a number of reviews concerning this topic were published [46,47,48].

Such extensive development of biosensors set the specific requirements for the transducers materials, namely their properties. Transducer materials, first of all, should have good biological affinity, enabling efficient immobilization of the biosensitive layer. Secondly, but not least, it should provide an intense output signal (electronic or optical, *etc*.) depending on the sensing type.

**Figure 5:**
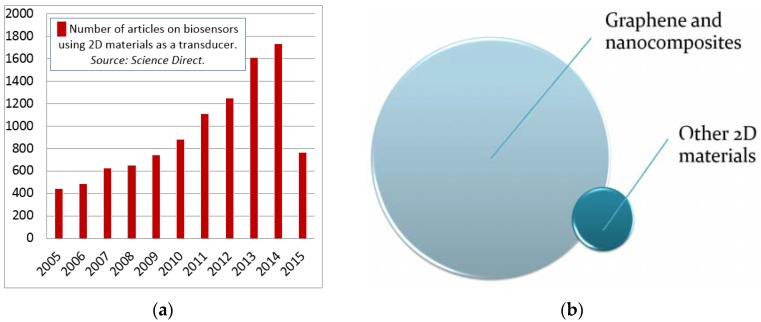
Dynamics of the reports on 2D materials application as a transducer material for biosensors during 2005–2015. Source: Science Direct (**a**). Among them, in 2014–2015: data on the biosensor articles reporting graphene and its nanocomposites as a transducer *vs.* non-graphene 2D materials (**b**).

Recent nanotechnology-oriented research provided plenty of novel material systems, appropriate for biosensors design (nanostructures, quantum dots (QDs), carbon nanotubes (CNT), graphene and, recently, other 2D materials). Being in fact the extreme case of surface science, 2D materials possess the highest surface-to-volume ratio. This feature makes them extremely prospective for sensors applications, where the interface occurring phenomena define the device performance. Therefore, the number of reports devoted to biosensors using 2D materials as a transducer has been constantly growing since the graphene discovery, as is evidenced by a simple search (Figure 5a). Apparently, such progress is due to extensive graphene development as a material, and only a small number of reports is devoted to biosensors based on non-graphene 2D materials (Figure 5b). However, it is noteworthy that the articles reporting the application of 2D non-graphene materials are most recent, being dated mainly at last two years ago. Several remarkable reviews have been recently published on this topic, reporting the application of graphene-like materials and graphene analogues in biomedical and biosensing applications [49,50]. Additionally, this can be explained as being due to the fact that 2D non-graphene materials have the following advantages over graphene in context of electronics and, hence, biosensor applications:

- Primarily, since graphene has a zero band gap, the transistors based on intrinsic graphene have a low on-to-off current ration, resulting in high standby power dissipation, which limits their real circuit application [51]. While 2D non-graphene materials have almost all of the necessary range of band gap values (Figure 2), they can be used for the design of a field effect transistor (FET) device. FET is characterized by high electron mobility and a high on-to-off ratio. Thus, integrating the 2D non-graphene material-based channel of FET with biosensing layers, one can expect the design of a complex biosensing device (FET biosensor). Such devices possess an extremely high sensitivity due to the enhancement of the interface-related phenomena and selectivity due to the immobilized biosensitive layers’ affinity.
- Another significant feature of 2D non-graphene materials is that unlike graphene or Si, many of them have either an intrinsic direct band gap in a bulk state or undergo the transition from indirect to direct semiconductors upon being scaled down to single layers [51]. This opens up their application as a transducer for biosensors of the optical type of detection, where their strong light-matter interaction can be influenced by the interface-related biological actions.
- Finally, it has to be noticed that among the various transducer materials that have been developed, nanostructured metal oxides are promising due to their exceptional optical and electrical properties that offer excellent prospects for the interfacing of biological recognition events with electronic or optical signal transduction and for the designing of a new generation of bioelectronics devices that may exhibit novel functions.

## 3. Application of 2D Non-Graphene Materials and 2D Nanostructures in Biosensor Design

Recently, there were several excellent reviews published, devoted to biosensors, based on graphene (e.g., [15]). However, the area of graphene analogues is practically undiscovered and not covered by any specific review. Therefore, we have focused our attention on the most recent reports on biosensors, based on 2D non-graphene materials. Interestingly, that report on the biosensoring properties of 2D non-graphene materials follows their development trend: most articles are devoted to molybdenum disulfide (MoS_2_) and tungsten disulfide (WS_2_) as the pioneering non-graphene materials. Later, this trend continued for other materials, including other dichalcogenides and chalcogenides (SnS_2_, CuS, *etc.*), and most recently by metal oxides (MnO_2_, ZnO).

The dominating detection principle of the biosensors reported is electrochemical, being marginally represented by optical types (see Figure 4). The electrochemical biosensors, based on non-graphene 2D materials, cover a range of the spectrum of analytes to be detected, such as glucose, dopamine, hydrogen peroxide and DNA [52].

**Figure 6:**
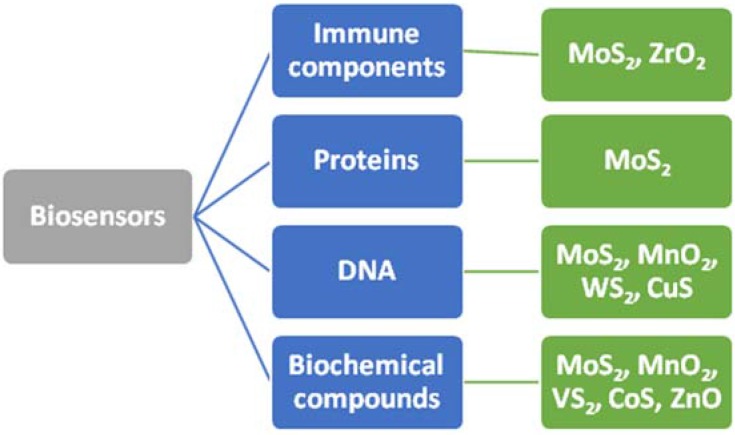
Demonstration of the ability of some 2D non-graphene material-based biosensors to be used for the detection or control of different biological substances.

We have summarized the reported 2D non-graphene material-based biosensors into several groups, depending on the analytes they were used to detect (Figure 6). As one can see, despite the fact that the materials are only at the sunrise of their application in biosensing technologies, they are able to cover a wide range of biological substances.

### 3.1. MoS_2_ Material for Electrochemical and Optical Biosensors

The MoS_2_ crystal consists of a metal Mo layer sandwiched between two S layers, with these triple layers stacking together to form a layered structure. It has been predicted that the layered MoS_2_ is expected to act as an excellent functional material, because the two-dimensional electron-electron correlations among Mo atoms would aid in enhancing the planar electric transportation properties. Indeed, the most dominating 2D non-graphene material in biosensor applications is unambiguously MoS_2_. This can be explained by its rather “mature” age, as well as good material stability in an ambient atmosphere (see Figure 1).

One of the most advanced MoS_2_ biosensor performance has been recently reported by Sarkar *et al.* [53], where the effect transistor (FETs) concept for biosensors design was elaborated. The authors emphasized that interest in biosensors based on FETS is stimulated by their highly desirable attributes, such as rapid electrical detection without the need for labeling the biomolecules, low power consumption, portability, inexpensive mass production and the possibility of on-chip integration of both sensor and measurement systems.

In a conventional FET used for digital applications, two electrodes (source and drain) are used to connect a semiconductor material, the so-called channel. Current flowing through the channel between the source and drain is electrostatically modulated by a third electrode called the gate, which is capacitively coupled through a dielectric layer covering the channel region.

**Figure 7:**
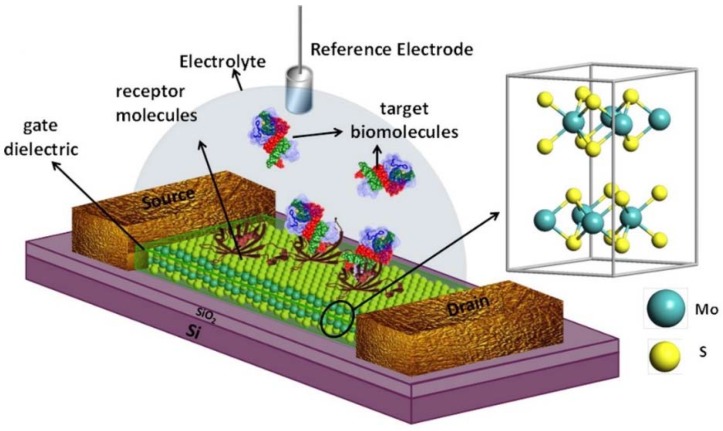
Schematic diagram of MoS_2_-based FET biosensor. For biosensing, the dielectric layer, covering the MoS_2_ channel, is functionalized with receptors for specifically capturing the target biomolecules. The charged biomolecules, after being captured, induce a gating effect, modulating the device current. An electrolyte gate is in the form of a reference electrode (Ag/AgCl) is used for applying bias to the electrolyte. Adapted with permission from [53]. Copyright 2014 American Chemical Society.

While in the case of an FET biosensor (Figure 7), the physical gate is removed and the dielectric layer is functionalized with a specific biosensitive layer for selectively capturing the desired target biomolecules, during the capture of the biomolecules, which are charged, a gating electrostatic effect is produced, which is then further transduced into a signal in the form of a change in the electrical characteristics of the FET, such as drain-to-source current or channel conductance [53].

The device, fabricated by Sarkar *et al.*, demonstrated extremely high sensitivity: a MoS_2_-based pH sensor demonstrated a sensitivity as high as 713 for a pH change by one unit along with efficient operation over a wide pH range (3–9). Via immobilization, the protein biotin, the ultra-sensitive and specific streptavidin protein, sensing was also achieved with a sensitivity of 196 even at 100 femto molar concentration [53].

Interestingly, the authors claim that graphene cannot compete with a MoS_2_-based FET biosensor, which surpasses the sensitivity of that based on graphene by more than 74-fold. Furthermore, MoS_2_, being highly flexible and having a transparent nature, can offer new opportunities in advanced diagnostics and medical prostheses. This unique fusion of desirable properties makes MoS_2_ a highly potential candidate for next-generation low-cost biosensors [53].

Narayanan *et al.* demonstrated the electrochemical enzymatic and non-enzymatic biosensing applications of ultrathin MoS_2_-based electrodes [54]. Atomically thin sheets of MoS_2_ were synthesized and isolated via solvent-assisted chemical exfoliation.

Firstly, the MoS_2_ sheets were studied using positively-charged hexamine ruthenium (III) chloride and negatively-charged ferricyanide/ferrocyanide redox probes for examining the charge-dependent electrochemical activities of the electrodes. An extensive study indicates that MoS_2_ electrodes can be extended to the selective detection of different biomolecules. In parallel, the ultrathin MoS_2_ sheet-based electrodes were employed for the electrochemical detection of such an important neurotransmitter as dopamine (DA), in the presence of ascorbic acid (AA). It is revealed, that MoS_2_ electrodes were capable of distinguishing the coexistence of the DA and the AA with an excellent stability.

The enzymatic detection of glucose was studied by immobilizing glucose oxidase on the MoS_2_ electrodes. It was concluded that the MoS_2_ surface is a favorable surface for enzyme accommodation: organic molecules can bind with the MoS_2_ surface efficiently, since their binding properties are greater than highly-oriented pyrolytic graphite or mica. Thus, even the simple application of MoS_2_ as an electrode opens up the possibility for highly sensitive enzymatic biosensing applications [54].

Another example of a label-free and ultra-sensitive electrochemical biosensor of DNA was demonstrated by *Wang et al.* [55], using thin-layer molybdenum disulfide (MoS_2_) nanosheets as a sensing platform. The thin-layer MoS_2_ nanosheets were prepared via a simple ultrasound exfoliation method from bulk MoS_2_. The authors postulate that this procedure allows the formation of MoS_2_ with enhanced electrochemical activity. It was shown that based on the high electrochemical activity and different affinity toward ssDNA *versus* dsDNA of the thin-layer MoS_2_ nanosheet sensing platform, the tlh gene sequence assay was performed label-freely for the concentrations from 1.0 × 10^−16^ M–1.0 × 10^−10^ M with a detection limit as low as 1.9 × 10^−17^ M. Due to the utilized MoS_2_, a viable alternative for DNA analysis was achieved, which has the priority in sensitivity, simplicity and costs. The authors emphasized also that the proposed sensing platform has good electrocatalytic activity and can be extended to detect more targets, such as guanine and adenine [55].

**Figure 8:**
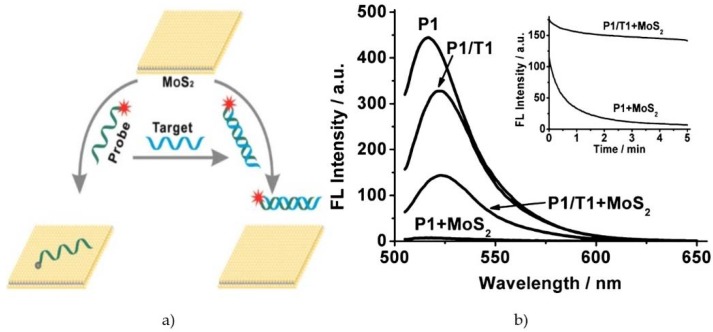
(**a**) Schematic illustration of the fluorometric DNA assay; (**b**) effect of MoS_2_ on the quenching intensity of the dye-labelled ssDNA probe P1 (*Homo sapiens* tumor suppression gene) and retained fluorescence for P1/T1. A single-layer MoS2 nanosheet exhibits a high fluorescence quenching ability and different affinity toward ssDNA *versus* dsDNA. The inset of (b) represents the quenching kinetics of the sensing process. Adapted with permission from [56]. Copyright 2013 American Chemical Society.

The optical type of biosensors utilizing MoS_2_ is mainly fluorescence-quenching-based devices. Such biosensors, comprising in fact homogeneous arrays for target molecules with fluorogenic probes, are becoming increasingly popular due to their inherent advantages, such as operation convenience, rapid binding kinetics and ease of automation. The probes by themselves usually contain a fluorophore and a quencher to form a Förster resonance energy transfer (FRET) pair, in which the distance-dependent fluorescence quenching is closely coupled with biomolecular recognition events. Zhu *et al.* demonstrated recently a simple and homogeneous assay format for DNA and small molecules by using single-layer MoS_2_-based fluorogenic nanoprobes. The authors presented the next ”mix-and-detect” strategy (Figure 8). Single-layer MoS_2_ can be considered as an ”S-Mo-S” sandwich structure, stacking a positively-charged molybdenum plane between two negatively-charged sulfur planes. MoS_2_ adsorbs a dye-labeled single-stranded DNA (ssDNA) probe via the van der Waals interaction between the nucleobases and the basal plane of MoS_2_ and then quenches the fluorescence of the dye. In contrast, when an ssDNA probe is hybridized with its complementary target DNA (since the nucleobases are buried between the dense negatively-charged helical phosphate backbones), the interaction between MoS_2_ and double-strained DNA is weaker; thus, the dye-labeled probe is away from the material surface, resulting in the retention of the fluorescence of the probe [56].

Thus, Zhu *et al.* revealed that a single-layer MoS_2_ nanosheet possesses high fluorescence quenching efficiency and different affinities toward ssDNA *versus* dsDNA. Inspired by these findings, the authors employed a MoS_2_ nanosheet as a sensing platform for the detection of DNA and small molecules. This mix-and-detect assay format is simple and can be finished within a few minutes. Importantly, the assay is homogeneous, because it occurs exclusively in the liquid phase, which makes it easy to automate or suitable for *in situ* detection [56].

Huang *et al.* developed a novel MoS_2_ nanosheet-based microfluidic biosensor for the ultra-sensitive detection of DNA. Compared to other nanomaterials, such as graphene, high concentration ultrathin MoS_2_ nanosheets can be readily synthesized on a large scale in aqueous solution and can be directly used to interact with DNA without further processing. Remarkably, MoS_2_ nanosheets are able to quench most of the fluorescence in a very short time (~min) and possess different affinities towards ssDNA *versus* dsDNA. The authors noticed that these properties of MoS_2_ make it perfect to be integrated with microfluidics. By using a high concentration MoS_2_ nanosheet solution uniformly mixed with the testing sample in zigzag-shaped microchannels, ssDNA and dsDNA can be easily and consistently distinguished within the range of ~min (more than 90% quenching efficiency was obtained within 1 min). This microfluidic biosensor can detect as low as 0.5 fmol target DNA, which is much lower than other similar nanoprobe-based fluorescence methods in bulk solution. The research conducted provides a simple and high throughput analysis method for rapid DNA screening [57].

Another example of the fluorescence quenching-based MoS_2_ biosensor is reported by Kong *et al.* [58], demonstrating a novel aptamer-functionalized MoS_2_ nanosheet fluorescent biosensor for the detection of prostate-specific antigen (PSA). Prostate-specific antigen (PSA) is a significant and the most widely-used biomarker for the early diagnosis of prostate cancer and its subsequent treatment. The principle of operation of the biosensing assay is the following: the binding of the aptamer to the target PSA induces a rigid aptamer structure, which makes the integration with the MoS_2_ nanosheet very weak. This results in the release of the aptamer probe from the nanosheet surface and restores the quenched fluorescence. The fabricated biosensor demonstrated high sensitivity and high selectivity with a detection limit for the PSA of 0.2 ng/mL. Later, the biosensor was further applied for the detection of PSA in human serum samples with satisfactory results. The foregoing indicates its promising application to real-life biological samples. The authors emphasized a higher fluorescence-quenching ability of MoS_2_ than graphene, when applied to a dye-labeled single-stranded DNA probe. Additionally, the simple design and rapid detection of PSA were reported as the advantages of this approach [58].

Finally, the biosensing assays comprising MoS_2_ were fabricated and tested for detection of heavy metals, particularly Ag ions [59]. Heavy metals are highly toxic and carcinogenic, even at a trace level, which can enter the environment due to increasing industrial activities. They are non-biodegradable and can accumulate in the food chain, posing a severe threat to the environment and human health. Among these heavy metal ions, silver ions (Ag^+^) have received substantial attention in recent years because the use of silver, silver nanoparticles and silver compounds has increased, and recent studies emphasized bioaccumulation and the potential negative impact of Ag^+^ on aquatic organisms. A single layer of MoS_2_ was used as the fluorescence quencher, and the operational principle was similar to that described above: FITC-labeled ssDNA was absorbed rapidly when approaching the surface of ultrathin MoS_2_ and was then quenched owing to charge transfer. An FITC-labeled Ag^+^-specific oligonucleotide, rich in cytosine, was employed as the fluorescent probe in sensing targets. The designed sensor demonstrated high fluorescence quenching efficiency within 5 min, excellent robustness, selectivity and sensitivity below the maximum limitation guided by the United States Environmental Protection Agency (EPA) and the World Health Organization (WHO). Further, this new Ag^+^ probe was demonstrated in monitoring Ag^+^ in lake water samples with satisfactory results [59].

However, not only MoS_2_ layers alone were used, but their combination with graphene or metal nanoparticles was reported to result in efficient transducer materials. Thus, Su *et al.* developed an electrochemical glucose biosensor by immobilizing glucose oxidase (GOx) on a glass carbon electrode that was modified with molybdenum disulfide (MoS_2_) nanosheets, decorated with gold nanoparticles (AuNPs). The synergistic effect the MoS_2_ nanosheets and the AuNPs resulted in excellent electrocatalytic activity. The electrochemical performance of the fabricated electrode was studied by the cyclic voltammetry, and it was revealed that the use of the AuNPs-decorated MoS_2_ nanocomposite accelerates the electron transfer from electrode to the immobilized enzyme. This enables the direct electrochemistry of GOx without any electron mediator. The fabricated sensor was able to detect glucose with a high sensitivity within the concentration range from 10–300 μM and down to levels as low as 2.8 μM. The authors acknowledge also the good reproducibility and long-term stability of the electrode, suggesting that it represents a promising tool for biological assays [60].

Nanocomposites based on MoS_2_, graphene (Gr) and horseradish peroxidase (HRP) were prepared by Song *et al.* [61]. It was demonstrated, that the native structure of the horseradish peroxidase is maintained after the assembly, implying good biocompatibility of MoS_2_-Gr nanocomposite. The fabricated biosensor based on HRP-MoS_2_-Gr composite displayed electrocatalytic activity to hydrogen peroxide (H_2_O_2_) with high sensitivity (~680 μA·mM^−1^·cm^−2^), a wide linear range (0.2 μM–1.1 mM), a low detection limit (0.05 μM) and a fast amperometric response. The biosensor also exhibited high selectivity, rather good stability and reproducibility. The authors attribute the obtained electrochemical properties to the good biocompatibility and electron transport efficiency of the MoS_2_-Gr nanocomposite and the efficient loading of HRP. It was suggested that the fabricated biosensor is potentially suitable for H_2_O_2_ analysis in environmental, pharmaceutical, food or industrial applications.

**Figure 9:**
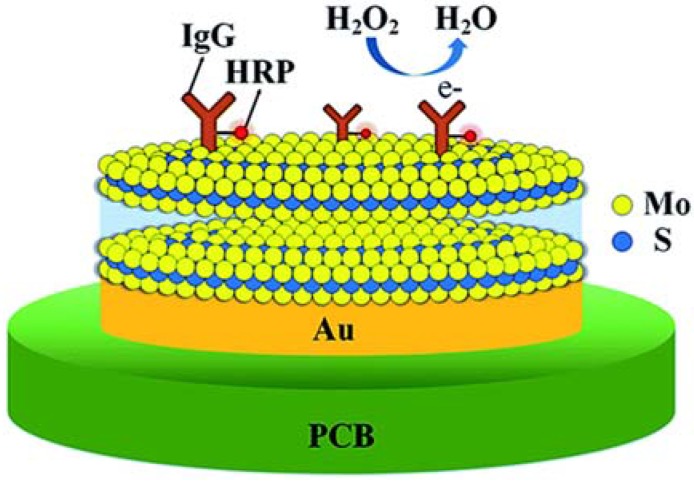
Schematic of the MoS_2_ biosensor device. For biosensing, the MoS_2_ is functionalized with receptors for specifically capturing target biomolecules. Image adapted from [62]; published by The Royal Society of Chemistry.

Kim *et al.* demonstrated *in situ* fabrication of the MoS_2_-based nanocomposite for biosensing applications. Thus, the MoS_2_ was grown by plasma-enhanced chemical vapor deposition (PECVD) on the Au layer, covering the polymeric printed circuit boards (PCB). The depositing Mo layer first was mixed with the Au, creating the Au-Mo composite structure. Then, the composite reacted with the H_2_S gas in Ar plasma, providing the nanocomposite coating. Via further immobilization of HRP-conjugated IgG on the Au electrode, modified with MoS_2_, the nanocomposite electrode was fabricated and utilized for sensing H_2_O_2_ (Figure 9). Trace H_2_O_2_ released from IgG-horseradish peroxidase was successfully detected in the linear range of 0–20 ng/mL [62].

Huang *et al.* reported a novel electrochemical sensor for the determination of bisphenol A (BPA) based on MoS_2_ and a chitosan-gold nanoparticles composite-modified electrode. First, flower-like MoS_2_ sheets were prepared by a simple hydrothermal method and had a lateral size of about 200 nm and a thickness of several nanometers. The sensing platform, was fabricated based on MoS_2_ and a chitosan (CS) mixture with Au nanoparticles (Au NPs), covering the glassy carbon electrode (GCE). Such a combination of AuNPs/MoS_2_/GCE caused the electrode to possess low background current, good conductivity and a large electro-active surface area. The fabricated electrochemical sensor was used for the determination of bisphenol A (BPA). BPA is a typical endocrine disruptor, which can increase cancer rate, decrease semen quality, reduce immune function and impair reproduction. Nevertheless, this chemical is still actively used in the chemical industry for the production of infant bottles, food packaging and canned soft drinks. The sensor showed an efficient electrocatalytic role for the oxidation of BPA, and the oxidation over potentials of BPA decreased significantly, which the peak current increased greatly compared to bare GCE and other modified electrodes. A good linear relationship between the oxidation peak current and BPA concentration was obtained in the range from 0.05–100 µM with a detection limit of 5.0 × 10^−9^ M, being followed by long-term sensing stability [63].

### 3.2. WS_2_

The application of WS_2_ for biosensors is somehow less reported in the literature than the MoS_2_, despite the materials’ similarity. Nevertheless, the biosensors of both the electrochemical and optical type were demonstrated, being based on both single WS_2_ sheets and their nanocomposites.

**Figure 10:**
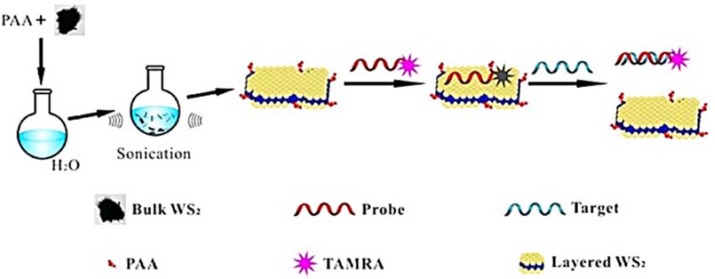
Schematic Illustration of fluorescence sensing of nucleic acid and protein with layered WS_2_ nanosheet as the quencher. Adapted with permission from [64]. PAA is polyacrylic acid; TAMRA is the TAMRA dyelabeled ssDNA probe P1: 5’-TAMRA-AGTCAGTGTGGAAAATCTCTAGC-3. Copyright 2014. American Chemical Society.

Thus, the application of WS_2_ as a platform in a fluorescence-quenching biosensor was recently reported by Yuan *et al.* [64]. The simple and straightforward synthesis route was proposed by a one-step sonication-assisted exfoliation method to prepare water-soluble WS_2_ nanosheets. The authors demonstrated that similarly to the case of MoS_2_, single-strand DNA (ssDNA) chains can be adsorbed on the WS_2_ nanosheet, leading to complete and fast quenching of a fluorescent dye tagged to the DNA chain upon reaction with the targeted analyte. The process of WS_2_ isolation and its surface functionalization with dye-tagged DNA is shown on Figure 10.

The adsorbed ssDNA is detachable from the nanosheet upon the interaction with other biomolecules, resulting in the restoration of the fluorescence. The 2D WS_2_ nanosheet thus acts as an efficient platform for assembling of bioprobes. Because of the extraordinarily high quenching efficiency, which is the synergic result of both excited-state energy transfer and static quenching, it is concluded that the WS_2_ platform provides minimal background and high sensitivity [64].

The electrochemical type of biosensor, using the nanocomposites comprising the WS_2_ sheets, was reported by Huang *et al.* [65]. The aptamer-based label-free electrochemical biosensor was used for the detection of 17b-estradiol. The aptamers immobilized on the glassy carbon electrode, modified by WS_2_ nanosheets and Au NPs through the Au-S interaction. Bovine serum albumin was used for blocking the free electrode surface. Via the addition of 17b-estradiol, the estradiol/aptamer complex on the electrode surface was created, resulting in the significant decrease in peak current. The change in the peak current is a sensor signal and has a good linear relationship with 17b-estradiol concentration for the range of 1.0 × 10^−11^–5.0 × 10^−9^ M, with a detection limit of 2.0 × 10^−12^ M. The biosensor was used in a real environment (in serum and water samples) and exhibited high sensitivity and reproducible analytical performance.

It has been concluded that the layered WS_2_ nanosheet/Au NPs nanocomposite coating can therefore act as an efficient electrochemical biosensing platform for the assembling of bio-probes and will be extended to other analytes, such as protein, DNA and RNA [65].

### 3.3. VS_2_

VS_2_ is one more candidate of layered materials that was reported to be used as a biosensor. Thus, Yin *et al.* [66] proposed a novel ultrasensitive sensing system for rapid fluorescence detection of cytochrome c (cyt c) by combining an aptamer-based bioassay with VS_2_ nanosheets. VS_2_ nanosheets with high fluorescence quenching ability were synthesized by the solution route. A cyt c-binding aptamer was tagged with the fluorescent dye carboxy fluorescein (FAM), acting as the probe. VS_2_ nanosheets were able to adsorb the probe and quench its fluorescence efficiently. However, the fluorescence of the probe was retained when it was incubated with cyt c and then mixed with VS_2_ nanosheet solution. The proposed sensing system shows high selectivity and sensitivity, giving a linear range of 0.75 nM–50 mM and a limit of detection of 0.50 nM [66].

Huang *et al.* reported the application of the VS_2_ nanocomposite with AuNPs as the electrochemical 17β-estradiol biosensor. First, the flower-like vanadium disulfide (VS_2_) was synthesized by a simple one-step hydrothermal process. Data from XRD, SEM and TEM demonstrated that the as-prepared VS_2_ product has an ordered nanosheet stacking flower structure, which is constructed with many irregular nanosheets as a petal-like structure with a thickness of several nanometers. The biosensor was constructed by immobilizing the aptamer on the surface of VS_2_ nanoflowers and an AuNP-modified glassy carbon electrode. Differential pulse voltammetry was applied, and a good linear relationship between the peak current and the logarithm of the 17β-estradiol concentration from 1.0 × 10^−11^–1.0 × 10^−8^ M was observed, with a detection limit of 1.0 × 10^−12^ M. The aptamer sensor was successfully applied for the determination of 17-estradiol in urine samples with recoveries of 92.0%–105.2%. It exhibited a high sensitivity and remarkable reproducible analytical performance. The reported results suggest the vitality and prospect of VS_2_ material in electrochemical biosensing and catalytic areas [67].

### 3.4. CoS

Cobalt sulfide nanosheets were recently reported by Huang *et al.* [68] in the development of a sensitive sensing platform for 17β-estradiol by combining the aptamer probe and hybridization reaction.

2D CoS was synthesized by a simple hydrothermal method with L-cysteine as the sulfur donor. The electrochemical aptamer biosensor was constructed by assembling a thiol group-tagged 17β-estradiol aptamer on CoS and gold nanoparticle (AuNPs)-modified electrode. Methylene blue was applied as a tracer, and a guanine-rich complementary DNA sequence was designed to bind with the unbound 17β-estradiol aptamer for signal amplification. The binding of guanine-rich DNA to the aptamer was inhibited when the aptamer captured 17β-estradiol. Using guanine-rich DNA in the assay greatly amplified the redox signal of methylene blue bound to the detection probe. The CoS/AuNPs nanocomposite coating formed on the biosensor surface was reported to be a good conductor for accelerating the electron transfer.

The biosensor demonstrated a high sensitivity of detection with the dynamic concentration range spanning from 10^−9^–10^−12^ M with a detection limit of 7.0 × 10^−13^ M. It is noteworthy also that the fabricated biosensor exhibited good selectivity toward 17β-estradiol, even when interferents were presented at 100-fold concentrations [68].

### 3.5. CuS

Huang *et al.* [69] reported the application of copper sulfide (CuS) nanosheets together with Au NPs as an efficient nanocomposite electrode for a novel DNA electrochemical biosensor. CuS nanosheets were synthesized with acetylene black (AB) incorporated via a simple solvothermal route assisted by ethylene glycol. The electrode surface was DNA labeled using 6-mercapto-1-hexane immobilized on the CuS-AB/Au nanoparticles through the Au-S interaction. After blocking with 6-mercapto-1-hexanol, the probe DNA was bound with the addition of target DNA to form the double-stranded structure on the electrode surface. This led to a significant decrease of the peak current of electrochemical indicator [Fe(CN)_6_]^3−/4−^. Under optimum conditions, the amperometric signals decrease linearly with the target DNA concentrations ranging from 0.1 pM–1 nM. The detection limit was observed as low as 20 fM with an excellent selectivity, acceptable stability and reproducibility.

The authors concluded that the good analytical performance is attributed to the synergistic effect of acetylene black and the unique microstructure of CuS nanosheets. This can open up new opportunities for sensitive and selective detection of specific sequence DNA and provide a promising platform for biosensor design for other biomolecular detection [69].

### 3.6. g-C_3_N_4_

Graphite-like carbon nitride (g-C_3_N_4_) is a new semiconductor material with p-conjugated graphitic planes formed by the sp^2^ hybridization of carbon and nitrogen. The prominent feature of this semiconductor is that it demonstrates the electrochemiluminescence (ECL). Probably the first report on the synthesis of g-C_3_N_4_ was by Zhang *et al.* [70]. In this study, the properties of ultrathin two-dimensional g-C_3_N_4_ nanosheets prepared by a “green” liquid exfoliation route from bulk g-C_3_N_4_ in water were investigated. It was found that 2D g-C_3_N_4_ demonstrated enhanced intrinsic photoabsorption and photoresponse compared to the bulk g-C_3_N_4_. High stability, good biocompatibility, superior physiochemical properties and large surface area have triggered interest in 2D g-C_3_N_4_ nanosheets as a material for ECL biosensors. Indeed, it was recently reported for biosensors based on 2D g-C_3_N_4_ for the detection of DNA [71], biothiols in biological fluids [72], heparin [73], 2,4,6-trinitrophenol (TNP) [74], concanavalin A [75], dopamine [76] and cancer cells [77].

### 3.7. BN

Hexagonal boron nitride (h*-*BN) is a novel semiconductor material with wide band gap (E_g_ = 5.2 eV), which makes it almost an insulator. h*-*BN is usually present in the form of flakes or sheets and has become widely used recently in the fields of electronics, catalysis and sensing. 2D nanosheets of BN consist of alternating boron and nitrogen atoms, representing an extra smooth surface. It has to be noticed that until now, only several applications of h-BN as a biosensor were reported, while most of them are still in the embryonic stage; while even the nanosheets allotropes—nanotubes of BN—were reported as promising nanotransducers for functionalization of biosensitive layers [78,79].

One of the first reports that demonstrated utilization of BN nanosheets was by Uosaki *et al.* [80]. The authors realized the electrocatalyst based on BN nanosheets on gold, thus demonstrating the ability of inert nanosheets to be functionalized for the oxygen reduction reaction. This discovery opened up new ways to design effective biocatalysts based on BN nanosheets and paving a novel route to electroanalysis [80].

Interestingly, that h-BN is initially hydrophobic, which makes it difficult to dissolve in water, and an additional surface treatment has to be applied. Xu *et al.* have utilized chitosan to increase the BN solubility in aqueous solution for further immobilization of catalase, thus designing the enzyme biosensor for the detection of forchlorfenuron. The fabricated sensor demonstrated linear sensitivity for the analyte concentration from 0.5–10 mM with a detection limit 0.07 μM [81].

BN was reported for the successful detection of hydrogen peroxide (H_2_O_2_), which is a significant compound involved in many chemical and biological processes. The electrochemical sensor, based on nanocomposites consisting of BN nanosheets and Au NPs, was reported before [82]. This paves the way for the detection of other compounds, which dissociate catalytically with creating H_2_O_2_ as a byproduct, like glucose or cholesterol [83], depending on the immobilized oxidase molecule.

Several other reports on biosensors based on BN are focused on the fabrication of a nanocomposite containing the BN nanosheets. Thus, the novel nanocomposite, formed by h-BN nanosheets and graphene quantum dots (GQDs) with green fluorescence, was reported. The sensor was proven to be an efficient platform for cell imaging, due to the nanocomposite’s strong fluorescent intensity, stability, water solubility, *etc.* The nanocomposite thus was reported as promising to contribute significantly to diagnostic or therapeutic needs, for drug delivery, *etc.* [84].

Another type of nanocomposite was made based on h-BN nanosheets and Au nanoparticles as a label and applied for an immunosensor for the detection of interleukin-6 by the fluorescence and electrochemistry approaches. The sensor demonstrated excellent performance, which the authors attributed to the high surface area and morphology for antibodies’ immobilization [85].

## 4. Application of 2D Oxide Nanostructures as Transducers for Biosensors

Another type of inorganic non-graphene 2D material is metal oxides, which have recently stimulated plenty of interest as immobilizing matrixes for biosensor development. Nanostructured oxides of metals, such as zinc, iron, cerium, tin, zirconium, titanium and magnesium, have been found to exhibit interesting nanomorphological, functional biocompatible, non-toxic and catalytic properties [20]. Among the various immobilizing matrixes that have been developed, such 2D oxide nanosheets have exceptional optical and electrical properties due to electron and phonon confinement, high surface-to-volume ratios, modified surface work function, high surface reaction activity, high catalytic efficiency and strong adsorption ability. For these reasons, oxides, such as TiO_2_, MoO_3_, WO_3_ and ZnO [51,86], have been used for immobilization of biomolecules, including enzymes, nucleic acids and antibodies.

### 4.1. MnO_2_

For the first time, a homogeneous FRET sensing protocol using a MnO_2_ nanosheet as the energy acceptor was proposed by Yuan *et al.* [87]. The biosensors based on fluorescence quenching were designed for ochratoxin A (OTA) as the sensing target. First, the OTA aptamers tagged to fluorophores were spontaneously assembled on the flat MnO_2_ surface. This resulted in the energy transfer from the fluorophore to MnO_2_ and the respective quenching of the fluorescence. Exposure of the aptamers with OTA molecules changes the conformation of the aptamers, which reduces the exposure of the nucleobases. As a consequence, the physisorption of the aptamers on MnO_2_ surface is weakened. Thus, the emission of the fluorophore is expected to be recovered, which enables the recognition and quantification of the target [87].

Different from those reported for the traditional two-dimensional nanosheets, a sensing mechanism was reported by Zhai *et al.* [88] for *in vivo* sensing of ascorbic acid (AA) in rat brain. Single-layer MnO_2_ nanosheets were used for suppressing the fluorescence of 7-hydroxycoumarin. The mechanism for the fluorescence suppression is attributed to a combination of an inner filter effect (IFE) and a static quenching effect (SQE). The combination of IFE and SQE leads to an exponential decay in fluorescence intensity of 7-hydroxycoumarin with increasing concentration of MnO_2_ nanosheets in solution. Such a property allows optimization of the concentration of MnO_2_ nanosheets in such a way that the addition of reductive analyte (e.g., AA) will to the greatest extent restore the MnO_2_ nanosheet-suppressed fluorescence of 7-hydroxycoumarin through the redox reaction between AA and MnO_2_ nanosheets. Compared to the turn-on fluorescent method through first decreasing the fluorescence to the lowest level by adding concentrated MnO_2_ nanosheets, the method demonstrated here possesses a higher sensitivity, lower limit of detection and wider linear range. Upon the use of ascorbate oxidase to achieve the selectivity for AA, the turn-on fluorescence method demonstrated can be used for *in vivo* sensing of AA in a simple, but reliable way [88].

He *et al.* reported a facile surfactant-templated synthetic strategy for water-dispersible nanoplatelets of MnO_2_ and then established it as a new biosensing platform for probing and recognizing biomolecular interactions in a homogeneous solution. The sensing strategy is based on the attachment of the ssDNA on MnO_2_, with the DNA strand interacting non-covalently with nano-MnO_2_ by the van der Waals force between nucleotide bases and the basal plane of MnO_2_. This platform possesses three excellent features: (i) MnO_2_ with an excellent dispersibility in water can be synthesized in great force at room temperature and used as a quencher without further processing; (ii) the MnO_2_-based biosensor is low cost and can finish the biomolecular assay within a few minutes; (iii) the biosensing strategy can be applied to other types of molecular probes by simply changing the sequences of the ssDNA to a specific target [89].

### 4.2. α-MoO_3_

Balendhran *et al.* reported a design of FET, using the 2D molybdenum trioxide (MoO_3_) material. As a protein model, bovine serum albumin was used. α-MoO_3_ nano-flakes, with the majority of nano-flake thicknesses being equal to or less than 2.8 nm, were stacked in a nanostructured film, forming the conduction channel. The FET demonstrated impressive response time (≤10 s). The authors explain this due to the high permittivity of the 2D α-MoO_3_ nano-flakes. It has been concluded that the 2D α-MoO_3_ system offers a competitive solution for future biosensing applications [90].

### 4.3. ZnO

Zinc oxide is a semiconducting material with plenty of advanced properties, such as high catalytic efficiency, biocompatibility, chemical stability in physiological environments and low toxicity. It possesses a high isoelectric point (IEP) of about 9.5, which makes it a rather prospective candidate for biosensing applications [91]. 2D ZnO is an expected crystal modification, where the original wurtzite crystal is transformed in the stacking of layers of Zn and O atoms, weakly related between the layers (so-called hexagonal film). It should be noticed that until now, 2D ZnO was only initially demonstrated in scales far away from those applicable for biosensing. Nevertheless, we focus our attention on the report of ZnO nano-flakes, which were in fact wurtzite ZnO, but at the same time extremely thin and demonstrated an unambiguous advantage over other morphologies, *i.e.*, bulk or nanostructures. Thus, Vabbina *et al.* reported label-free, highly sensitive and selective electrochemical immunosensors based on 2D ZnO nano-flakes (ZnO-NFs) which were synthesized on Au-coated substrates using a simple one-step sonochemical approach. Selective detection of cortisol using cyclic voltammetry (CV) is achieved by immobilizing anti-cortisol antibody (Anti-Cab) on the ZnO nanostructures (NSs). 2D ZnO-NFs provide unique sensing advantages over bulk materials. 2D-NSs with a large area in the polarized (0001) plane and a high surface charge density could promote higher Anti-Cab loading and, thus, better sensing performance. Beside a large surface area, ZnO-NSs also exhibit higher chemical stability, high catalytic activity and biocompatibility. The measured sensing parameters are in the physiological range with a sensitivity of 7.74 mA/M with the lowest detection limit of 1 pM, which is 100-times better than the conventional enzyme-linked immunosorbent immunoassay (ELISA). ZnO-NS-based cortisol immunosensors were tested on human saliva samples, and the performance were validated with a conventional (ELISA) method, which exhibits a remarkable correlation. The developed sensors can be integrated with a microfluidic system and a miniaturized potentiostat for point-of-care cortisol detection, and such a developed protocol can be used in personalized health monitoring/diagnostics [92].

### 4.4. CuO

Copper oxide being a narrow gap semiconductor with *p*-type conductivity (*E_g_* = 1.2 eV in bulk crystal) has been used in many electronic applications and sensors. Such a metal oxide can have many well-defined nanostructures with different dimensionalities, possessing unique electronic and optical properties. For instance, due to the size confinement effect, the 2D CuO has an enhanced optical band gap (*E_g_* = 2.15 eV) [93]. Recently, 2D CuO has been reported as a promising material for the development of non-enzymatic sensors [94,95]. Most of the reports on 2D CuO up to now deal with an investigation of the properties of nano-leaves of copper oxide grown by chemical methods [96,97]. In particular, Bhattacharjee [96,97] reported the successful synthesis of 2D CuO nano-leaves with average dimensions of ~350–450 nm in length and ~60–90 nm in width by the green method using NaOH and L-arginine. Zhao *et al.* [94] designed a non-enzymatic glucose sensor based on 2D CuO nano-leaves. These authors showed the good electrocatalytic activity of the 2D CuO-containing electrode. The observed enhancement of peak current for the oxidation of glucose was attributed to (i) the increase in the electroactive surface area and (ii) the electron transfer ability of the electrode based on 2D CuO nano-leaves. Sun *et al.* [95] have studied the glucose sensors based on 2D hierarchical nanoporous CuO ribbons. It was reported that the designed sensors demonstrate a high sensitivity of 2241 μA·mM^−1^·cm^−2^, a fast response time of ∼2 s, a relatively wide linear dynamic range of 0.1–4.0 mM, a low detection limit of 50 nM and good anti-interference ability.

## 5. Conclusions and Outlook

It is revealed that the family of 2D non-graphene inorganic materials is undeniably forthcoming for the application as a transducer material in biosensors. This is indirectly proven by the growing research interest, reflected in a rising number of papers during the last few years. The scientific reports clearly demonstrate the certain advantages of 2D non-graphene materials over graphene (direct band gap in 2D *vs.* no band gap in graphene) or bulk crystals (the highest surface-to-volume ratio in 2D). Using the 2D non-graphene materials, the biosensors can be designed for different types, mainly electrochemical (where the 2D material is a regular electrode or serves as a channel in FET design) or optical (by quenching the fluorescence or retaining it via aptamers use).

Thus, the biosensors based on 2D non-graphene materials demonstrate a unique combination of high sensitivity, selectivity and dynamic characteristics (response and recovery time). It is shown that many different biological agents can be successfully immobilized on the surface of 2D non-graphene materials, providing sensing ability for a wide range of biological targets, as is demonstrated in Table 1. It is noteworthy that the potential of 2D non-graphene materials can be further extended via the fabrication of nanocomposites with graphene or noble metal nanoparticles.

**Table 1:** Example of non-graphene 2D materials’ application in different types of biosensors (2014/2015).

2D	Detection Type	Purpose	Sensitivity: Detection Range and Threshold	Comment	Reference
**MoS_2_**	electro-chemical	Determination of glucose	2.8 μM–300 μM	Biosensor was developed by immobilizing glucose oxidase (GOx) on a glass carbon electrode that was modified with MoS_2_ nanosheets that were decorated with Au NPs	[60]
	electro-chemical	Detection of dopamine	1.0 mM DA/pH 7.4	MoS_2_ sheet-based electrodes were employed for the electrochemical detection of an important neurotransmitter, namely dopamine (DA), in the presence of ascorbic acid (AA)	[54]
	FET	Detection of proteins	713 for a pH change of 1 unit	Biosensors based on field-effect transistors (FETs); specific detection of protein is also demonstrated, and an extremely high sensitivity of 196 was achieved, even at a 100 femtomolar concentration	[53]
	fluorescent	Detection of Ag	25 mg/mL	The developed sensor with high sensitivity and selectivity may be an alternative method for Ag ion detection in lake water samples and other applications	[59]
	fluorescent, microfluidic	Fluorescent DNA detection	0.2 µL	MoS_2_ nanosheets are able to quench most of the fluorescence in a very short time (~min) and possess different affinities towards ssDNA *versus* dsDNA	[57]
	electro-chemical	Immobilization horseradish peroxidase conjugated IgG	0–20 ng/mL	The cyclic voltammetry results showed that the sensor of Au-MoS_2_ conjugated with IgG-HRP thus exhibited excellent analytical responses to H_2_O_2_ with a wide linear range	[62]
	fluorescent	Detection of prostate specific antigen	0.2 ng/mL	The binding of the aptamer to the target PSA induces a rigid aptamer structure, which makes the integration with the MoS_2_ nanosheet very weak	[58]
	electro-chemical	DNA analysis	1.0 × 10^−16^–1.0 × 10^−10^ M	The tlh gene sequence assay can be performed label-freely with a detection limit of 1.9 × 10^−17^ M	[55]
	electro-chemical	Determination of bisphenol A	0.05–100 mM, (5.0 × 10^−9^ M)	Biosensor based on MoS_2_ and chitosan-gold nanoparticle composite-modified electrode	[63]
**MnO_2_**	fluorescent	In vivo sensing of ascorbic acid (AA)	2.7–25.9 mM^−1^	The authors investigate the mechanism of single-layer MnO_2_ nanosheets suppressing fluorescence of 7-β hydroxycoumarin	[89]
	fluorescent	DNA hybridization	0–5 nM	Probing DNA hybridization and aptamer-target interactions in a homogeneous solution	[90]
**VS_2_**	fluorescent	Detection of cytochrome c	0.75 nM–50 mM	VS_2_ nanosheets with a high fluorescence quenching ability were synthesized by the solution route	[66]
	electro-chemical	Determination of 17β-estradiol	1.0 × 10^−11^–1.0 × 10^−8^ M (1.0 × 10^−12^ M)	VS_2_ nanoflowers and gold nanoparticle-modified glassy carbon electrode	[67]
**WS_2_**	fluorescent	Platform for biosensing (ssDNA)	1−80 ng/mL	The adsorbed ssDNA is detachable from the nanosheet upon the interaction with other biomolecules, resulting in the restoration of the fluorescence	[88]
	electro-chemical	Determination of 17β-estradiol	1 × 10^−11^–5.0 × 10^−9^ M (2.0 × 10^−12^ M)	Aptamers immobilized on the WS_2_ nanosheets/AuNP-modified glassy carbon electrode	[65]
**CoS**	electro-chemical	Determination of 17β-estradiol	1.0 × 10^−9^–1.0 × 10^−12^ M (7.0 × 10^−13^ M)	Thiol group tagged 17β-estradiol aptamer on CoS and AuNP-modified electrode	[68]
**CuS**	electro-chemical	Detection of DNA	0.1 pM−1 nM (20 fM)	DNA labeled at 5 end using 6-mercapto-1-hexhane immobilized on the CuS- acetylene black (AB)/Au-modified electrode	[69]
**h-BN**	electro-chemical	Detection of forchlorfenuron	0.5 to 10 mM (0.07 μM)	The fabricated enzyme-based sensor demonstrated linear sensitivity for range 0.5–10 mM with a detection limit 0.07 μM	[81]
**CuO**	electro-chemical	Glucose	2241 μA·mM^−1^·cm^−2^, 0.1–4 mM	Glucose level was detected by a fast (~2 s) and precise technique	[96]
**ZnO**	electro-chemical	Detection of cortisol	7.74 mA/M	Immunosensor based on 2D ZnO nano-flakes synthesized on Au-coated substrates	[93]

Chalcogenides are the dominant materials among the non-graphene 2D, reported in biosensors’ design (MoS_2_, WS_2_, VS_2_, CoS, CuS). Specifically, MoS_2_ is the most reported non-graphene 2D material in the biosensing area. This may be explained by its several advantages, including comparatively simple synthesis procedure and possibility of direct (CVD) growth. Another important advantage of MoS_2_ for biosensors is that it has polarized planes, which favors the van der Waals interaction and promotes the biosensitive layers’ immobilization, for both enzyme-based and non-enzyme electrochemical biosensors. The realization of the FET-based biosensing device with MoS_2_ as a channel results in obtaining a highly sensitive, single-molecule detection compatible sensor. Eventually, the performance/sensitivity of the MoS_2_-based FET biosensor was reported to be 74-fold better than that one of the graphene-based one. In the optical type of biosensors, MoS_2_ has high fluorescence quenching ability, good detection limits and fast response time. Via functionalization of MoS_2_ by aptamers, the optical biosensor demonstrates high sensitivity with a simple design and low cost. Finally, nanocomposites containing MoS_2_ together with graphene and/or metal nanoparticles were reported to be efficient transducers for the electrochemical type of biosensors of a wide range of analytes.

Among the 2D nitride materials, g-C_3_N_4_ was reported to have intense electrochemiluminescence, which can be used for the detection of DNA, cancer cells, dopamine, concanavalin, heparin and biothiols; while h-BN nanosheets can be functionalized for electrocatalysis and further detection of hydrogen peroxide, forchlorfenuron, interleukin-6, *etc.*

While 2D metal oxides are represented mainly by MnO_2_, MoO_3_, CuO and, lately, ZnO, 2D oxides are explicitly promising due to their exceptional optical and electrical properties, which offer excellent prospects for interfacing of biological recognition events with electronic or optical signal transduction and for designing of a new generation of bioelectronics devices. Specifically, MnO_2_ is a transition-metal oxide with good water-solubility, excellent biocompatibility and easy modification, which is important for sensor fabrication; while CuO has been reported as a promising material for the development of non-enzymatic sensors, mainly glucose.

Another intriguing oxide is ZnO, due to its wide band gap, high catalytic efficiency, biocompatibility and chemical stability in physiological environments, low toxicity and a high isoelectric point (IEP) of about 9.5. All the above makes 2D ZnO therefore extremely promising for biosensing applications. This, however, is still compromised by its practical unattainability, which will be an exciting materials researcher’s task for the next few years. Finally, we would like to conclude that the present tendency of growing the 2D materials library will result in the appearance of new candidates, which will definitely join the biosensing area soon.

## References

[1] Novoselov K.S., Geim A.K., Morozov S.V., Jiang D., Zhang Y., Dubonos S.V., Grigorieva I.V., Firsov A.A. (2004). Electricfield Effect in Atomically Thin Carbon films. Science.

[2] Stoller M.D., Park S.J., Zhu Y.W., An J.H., Ruoff R.S. (2008). Graphene-Based Ultracapacitors. Nano Lett..

[3] Stankovich S., Dikin D.A., Piner R.D., Kohlhaas K.A., Kleinhammes A., Jia Y., Wu Y., Nguyen S.T., Ruoff R.S. (2007). Synthesis of Graphene-Based Nanosheets via Chemical Reduction of Exfoliated Graphite Oxide. Carbon.

[4] Yoo J.J., Balakrishnan K., Huang J.S., Meunier V., Sumpter B.G., Srivastava A., Conway M., Reddy A.L.M., Yu J., Vajtai R. (2011). Ultrathin Planar Graphene Supercapacitors. Nano Lett..

[5] Wu Z.S., Ren W.C., Gao L.B., Liu B.L., Jiang C.B., Cheng H.M. (2009). Synthesis of High-Quality Graphene with a Pre-Determined Number of Layers. Carbon.

[6] Lee C., Wei X.D., Kysar J.W., Hone J. (2008). Measurement of the Elastic Properties and Intrinsic Strength of Monolayer Graphene. Science.

[7] Nair R.R., Blake P., Grigorenko A.N., Novoselov K.S., Booth T.J., Stauber T., Peres N. (2008). Fine Structure Constant Defines Visual Transparency of Graphene. Science.

[8] Ghosh S., Calizo I., Teweldebrhan D., Pokatilov E.P., Nika D.L., Balandin A.A. (2008). Extremely High Thermal Conductivity of Graphene: Prospects for Thermal Management Applications in Nanoelectronic Circuits. Appl. Phys. Lett..

[9] Chen Z., Yu D.S., Xiong W., Liu P.P., Liu Y., Dai L.M. (2014). Graphene-Based Nanowire Supercapacitors. Langumuir.

[10] Qian Y., Lu S.B., Gao F.L. (2011). Synthesis of Manganese Dioxide/Reduced Graphene Oxide Composites with Excellent Electrocatalytic Activity Toward Reduction of Oxygen. Mater. Lett..

[11] Shao Y.Y., Wang J., Wu H., Liu J., Aksay I.A., Lin Y.H. (2010). Graphene Based Electrochemical Sensors and Biosensors: A Review. Electroanalysis.

[12] Ferrari A.C., Bonaccorso F., Falko V., Novoselov K.S., Roche S., Bøggild P., Borini S., Koppens F., Palermo V., Pugno N. (2014). Science and technology roadmap for graphene, related two-dimensional crystals, and hybrid systems. Nanoscale.

[13] Butler S.Z., Hollen S.M., Cao L., Cui Y., Gupta J., Gutiérrez H.R., Heinz T.F., Hong S.S., Huang J., Ismach A.F. (2013). Progress, challenges, and opportunities in two-dimensional materials beyond graphene. ACS Nano.

[14] Li L., Yu Y., Ye G.J., Ge Q., Ou X., Wu H., Feng D., Chen X.H., Zhang Y. (2014). Black phosphorus field-effect transistors. Nat. Nanotechnol..

[15] Zhu C., Du D., Lin Y. (2015). Graphene and graphene-like 2D materials for optical biosensing and bioimaging: A review. 2D Mater..

[16] Geim A.K., Grigorieva I.V. (2013). Van der Waals heterostructures. Nature.

[17] Gupta A., Sakthivel T., Seal S. (2015). Recent development in 2D materials beyond graphene. Prog. Mater. Sci..

[18] Mas-Ballesté R., Gómez-Navarro C., Gómez-Herrero J., Zamora F. (2011). 2D materials: To graphene and beyond. Nanoscale.

[19] North S.H., Lock E.H. (2010). Critical aspects of biointerface design and their impact on biosensor development. Anal. Bioanal. Chem..

[20] Solanki P.R., Kaushik A. (2011). Nanostructured metal oxide-based biosensors. NPG Asia Mater..

[21] Ronkainen N.J., Halsall H.B., Heineman W.R. (2010). Electrochemical biosensors. Chem. Soc. Rev..

[22] Zhu C., Yang G., Li H., Du D., Lin Y. (2015). Electrochemical sensors and biosensors based on nanomaterials and nanostructures. Anal. Chem..

[23] Walcarius A., Minteer S.D., Wang J., Lin Y., Merkoçi A. (2013). Nanomaterials for bio-functionalized electrodes: Recent trends. J. Mater. Chem. B.

[24] Gea X., Asiri A.M., Du D., Wen W., Wang S., Lin Y. (2014). Nanomaterial-enhanced paper-based biosensors. Trends Anal. Chem..

[25] Ravindra N.M., Prodan C. (2007). Advances in the manufacturing, types, and applications of biosensors. JOM.

[26] Turner D.C., Chang C.Y., Fang K., Brandow S.L., Murphy D.B. (1995). Selective adhesion of functional microtubules to patterned silane surfaces. Biophys. J..

[27] Clark L.C., Lyons C. (1962). Electrode systems for continuous monitoring in cardiovascular surgery. Ann. N. Y. Acad. Sci..

[28] Renneberg R., Pfeiffer D., Lisdat F., Wilson G., Wollenberger U., Ligler F., Turner A.P.F. (2008). Frieder Scheller and the short history of biosensors. Adv. Biochem. Eng. Biotechnol..

[29] Scognamiglio V., Pezzotti G., Pezzotti I., Cano J., Buonasera K., Giannini D., Giardi M.T. (2010). Biosensors for effective environmental and agrifood protection and commercialization: From research to market. Microchim. Acta.

[30] Scheller F.W., Wollenberger U., Warsinke A., Lisdat F. (2001). Research and development in biosensors. Curr. Opin. Biotechnol..

[31] Teng Y., Zhang X., Fu Y., Liu H., Wang Z., Jin L., Zhang W. (2011). Optimized ferrocene-functionalized ZnO nanorods for signal amplification in electrochemical immunoassay of *Escherichia Coli*. Biosens. Bioelectron..

[32] Queiros R.B., Noronha J.P., Marques P.V.S., Sales M.G.F., Voudouris K. (2012). Emerging (bio)sensing technology for assessing and monitoring freshwater contamination—Methods and applications. Ecological Water Quality—Water Treatment and Reuse.

[33] Ansari A.A., Kaushik A., Solanki P.R., Malhotr B.D. (2010). Nanostructured zinc oxide platform for mycotoxin detection. Bioelectrochem.

[34] Soldatkin A.P., Volotovsky V., El’skaya A.V., Jaffrezic-Renault N., Martelet C. (2000). Improvement of urease based biosensor characteristics using additional layers of charged polymers. Anal. Chim. Acta.

[35] Ilangovan R., Daniel D., Krastanov A., Zachariah C., Elizabeth R. (2006). Enzyme based biosensor for heavy metal ions determination. Biotechnol. Biotechnol. Eq..

[36] Domínguez-Renedo O., Alonso-Lomillo M.A., Arcos-Martínez M.J. (2013). Determination of metals based on electrochemical biosensors. Crit. Rev. Env. Sci. Technol..

[37] Dhull V., Gahlaut A., Dilbaghi N., Hooda V. (2013). Acetylcholinesterase biosensors for electrochemical detection of organophosphorus compounds: A review. Biochem. Res. Int..

[38] Tusa J.K., He H. (2005). Critical care analyzer with fluorescent optical chemosensors for blood analytes. J. Mater. Chem..

[39] Osaka T., Komaba S., Seyama M., Tanabe K. (1996). High-sensitivity urea sensor based on the composite film of electroinactive polypyrrole with polyion complex. Sens. Actuators B Chem..

[40] Jijun T., Jie H., Zhongchao H., Min P., Yuquan C. A novel lactate biosensor. Proceedings of the 27th Annual International Conference of the IEEE Engineering in Medicine and Biology Society.

[41] Heinemann L. (2009). Continuous glucose monitoring and clinical trials. J. Diabetes Sci. Technol..

[42] Fojta M. (2002). Electrochemical sensors for DNA interactions and damage. Electroanalysis.

[43] Hang T.C., Guiseppi-Elie A. (2004). Frequency dependent and surface characterization of DNA immobilization and hybridization. Biosens. Bioelectron..

[44] Hofmann U., Michaelis S., Winckler T., Wegener J., Feller K.H. (2013). A whole-cell biosensor as *in vitro* alternative to skin irritation tests. Biosens. Bioelectron..

[45] Monosik R., Stredansky M., Strurdik E. (2012). Application of electrochemical biosensor in clinical diagnosis in clinical diagnosis. J. Clin. Lab. Anal..

[46] Kaushik A., Tiwari S., Jayant R.D., Marty A., Nair M. (2016). Towards detection and diagnosis of Ebola virus disease at point-of-care. Biosens. Bioelectron..

[47] Kaushik A., Yndart A., Jayant R.D., Sagar V., Atluri V., Bhansali S., Nair M. (2015). Electrochemical sensing method for point-of-care cortisol detection in human immunodeficiency virus-infected patients. Int. J. Nanomed..

[48] Kaushik A., Vasudev A., Arya S.K., Pasha S.K., Bhansali S. (2015). Recent advances in cortisol sensing technologies for point-of-care application. Biosens. Bioelectron..

[49] Yang G., Zhu C., Du D., Zhu J., Lin Y. (2015). Graphene-like two-dimensional layered nanomaterials: Applications in biosensors and nanomedicine. Nanoscale.

[50] Chen Y., Tan C., Zhang H., Wang L. (2015). Two-dimensional graphene analogues for biomedical applications. Chem. Soc. Rev..

[51] Minoru O., Takayoshi S. (2012). Two-Dimensional Dielectric Nanosheets: Novel Nanoelectronics from Nanocrystal Building Blocks. Adv. Mater..

[52] Late D.J., Rout C.S. (2015). A Perspective on Atomically Thin 2D Inorganic Layered Materials for Biosensor. J. Nanomed. Res..

[53] Sarkar D., Liu W., Xie X., Anselmo A.C., Mitragotri S., Banerjee K. (2014). MoS_2_ Field-Effect Transistor for Next-Generation Label-Free Biosensors. ACS Nano.

[54] Narayanan T.N., Vusa C.S.R., Alwarappan S. (2014). Erratum: Selective and Efficient Electrochemical Biosensing of Ultrathin Molybdenum Disulfide Sheets. Nanotechnology.

[55] Wang X., Nan F., Zhao J., Yang T., Ge T., Jiao T.A. (2015). Label-Free Ultrasensitive Electrochemical DNA Sensor Based on Thin-Layer MoS_2_ Nanosheets with high Electrochemical Activity. Biosens. Bioelectron..

[56] Zhu C., Zeng Z., Li H., Li F., Fan C., Zhang H. (2013). Single-Layer MoS_2_-Based Nanoprobes for Homogeneous Detection of Biomolecules. J. Am. Chem. Soc..

[57] Huang Y., Shi Y., Yang H.Y., Ai Y. (2015). A Novel Single-Layered MoS_2_ Nanosheet Based Microfluidic Biosensor for Ultrasensitive Detection of DNA. Nanoscale.

[58] Kong R.-M., Ding L., Wang Z., You J., Qu F. (2015). A Novel Aptamer-Functionalized MoS_2_ Nanosheet Fluorescent Biosensor for Sensitive Detection of Prostate Specific Antigen. Anal. Bioanal. Chem..

[59] Mao K., Wu Z., Chen Y., Zhou X., Shen A., Hu J. (2015). A Novel Biosensor Based on Single-Layer MoS_2_ Nanosheets for Detection of Ag. Talanta.

[60] Su S., Sun H., Xu F., Yuwen L., Fan C., Wang L. (2014). Direct Electrochemistry of Glucose Oxidase and a Biosensor for Glucose Based on a Glass Carbon Electrode Modified with MoS_2_ Nanosheets Decorated with Gold Nanoparticles. Microchim. Acta.

[61] Song H., Ni Y., Kokotc S. (2014). Investigations of an electrochemical platform based on the layered MoS_2_-graphene and horseradish peroxidase nanocomposite for direct electrochemistry and electrocatalysis. Biosens. Bioelectron..

[62] Kim H.-U., Kim H., Ahn C., Kulkarni A., Jeon M., Yeom G.Y., Lee M.-H., Kim T. (2015). In Situ Synthesis of MoS_2_ on a Polymer Based Gold Electrode Platform and its Application in Electrochemical Biosensing. RSC Adv..

[63] Huang K.-J., Liu Y.-J., Liu Y.-M., Wang L.-L. (2014). Molybdenum Disulfide Nanoflower-Chitosan-Au Nanoparticles Composites Based Electrochemical Sensing Platform for Bisphenol A Determination. J. Hazard. Mater..

[64] Yuan Y., Li R., Liu Z. (2014). Establishing Water-Soluble Layered WS_2_ Nanosheet as a Platform for Biosensing. Anal. Chem..

[65] Huang K.-J., Liu Y.-J., Shi G.-W., Zhang J.-Z., Liu Y.-M. (2014). A Novel Aptamer Sensor Based on Layered Tungsten Disulfide Nanosheets and Au Nanoparticles Amplification for 17β-Estradiol Detection. Anal. Methods.

[66] Yin X., Cai J., Feng H., Wu Z., Zou J., Cai Q. (2015). A Novel VS_2_ Nanosheet-Based Biosensor for Rapid Fluorescence Detection of Cytochrome C. New J. Chem..

[67] Huang K.-J., Liu Y.-J., Shi G.-W., Yang X.-R., Liu Y.-M. (2014). Label-Free Aptamer Sensor for 17β-Estradiol Based on Vanadium Disulfide Nanoflowers and Au Nanoparticles. Sens. Actuators B.

[68] Huang K.J., Liu Y.J., Zhang J.Z., Cao T., Liu Y.M. (2015). Aptamer/Au Nanoparticles/Cobalt Sulfide Nanosheets Biosensor for 17β-Estradiol Detection Using a Guanine-Rich Complementary DNA Sequence for Signal Amplification. Biosens. Bioelectron..

[69] Huang K.J., Liu Y.J., Zhang J.Z., Liu Y.M. (2015). A Sequence-Specific DNA Electrochemical Sensor Based on Acetylene Black Incorporated Two-Dimensional CuS Nanosheets and Gold Nanoparticles. Sens. Actuators B Chem..

[70] Zhang X.D., Xie X., Wang H., Zhang J.-J., Pan B.-C., Xie Y. (2013). Enhanced Photoresponsive Ultrathin Graphitic-Phase C3N4 Nanosheets for Bioimaging. J. Am. Chem. Soc..

[71] Wang Q.B., Wang W., Lei J.P., Xu N., Gao F.L., Ju H.X. (2013). Fluorescence Quenching of Carbon Nitride Nanosheet through Its Interaction with DNA for Versatile Fluorescence Sensing. Anal. Chem..

[72] Tang Y., Song H., Su Y., Lv Y. (2013). Turn-on persistent luminescence probe based on graphitic carbon nitride for imaging detection of biothiols in biological fluids. Anal. Chem..

[73] Ma T.Y., Tang Y., Dai S., Qiao S.Z. (2014). Proton-Functionalized Two-Dimensional Graphitic Carbon Nitride Nanosheet: An Excellent Metal-/Label-Free Biosensing Platform. Small.

[74] Rong M., Lin L., Song X., Zhao T., Zhong Y., Yan J., Wang Y., Chen X. (2015). A label-free fluorescence sensing approach for selective and sensitive detection of 2,4,6-trinitrophenol (TNP) in aqueous solution using graphitic carbon nitride nanosheets. Anal. Chem..

[75] Ou X., Tan X., Liu X., Lu Q., Chen S., Wei S. (2015). A signal-on electrochemiluminescence biosensor for detecting Con A using phenoxy dextran-graphite-like carbon nitride as signal probe. Biosens. Bioelectron..

[76] Liu Y., Wang Q., Lei J., Hao Q., Wang W., Ju H. (2014). Anodic electrochemiluminescence of graphitic-phase C3N4 nanosheets for sensitive biosensing. Talanta.

[77] Wang Y.Z., Hao N., Feng Q.M., Shi H.W., Xu J.J., Chen H.Y. (2016). A ratiometric electrochemiluminescence detection for cancer cells using g-C3N4 nanosheets and Ag–PAMAM–luminol nanocomposites. Biosens. Bioelectron..

[78] Zhi C.Y., Bando Y., Tang C.C., Huang Q., Golberg D. (2008). Boron nitride nanotubes: Functionalization and composites. J. Mater. Chem..

[79] Ciofani G., Danti S., Genchi G.G., Mazzolai B., Mattoli V. (2013). Boron Nitride Nanotubes: Biocompatibility and Potential Spill-Over in Nanomedicine. Small.

[80] Uosaki K., Elumalai G., Noguchi H., Masuda T., Lyalin A., Nakayama A., Taketsugu T. (2014). Boron Nitride Nanosheet on Gold as an Electrocatalyst for Oxygen Reduction Reaction: Theoretical Suggestion and Experimental Proof. J. Am. Chem. Soc..

[81] Xu Q., Cai L., Zhao H., Tang J., Shen Y., Hu X., Zeng H. (2015). Forchlorfenuron detection based on its inhibitory effect towards catalase immobilized on boron nitride substrate. Biosens. Bioelectron..

[82] Yang G., Abulizi A., Zhu J. (2014). Sonochemical fabrication of gold nanoparticles-boron nitride sheets nanocomposites for enzymeless hydrogen peroxide detection. Ultrason. Sonochem..

[83] Sodzel D., Khranovskyy V., Beni V., Turner A.P.F., Viter R., Eriksson M.O., Holtz P.-O., Janot J.-M., Bechelany M., Balme S. (2015). Continuous sensing of hydrogen peroxide and glucose via quenching of the UV and visible luminescence of ZnO nanoparticles. Michrochim. Acta.

[84] Peng J., Wang S., Zhang P., Jiang L., Shi J., Zhu J. (2013). Fabrication of Graphene Quantum Dots and Hexagonal Boron Nitride Nanocomposites for Fluorescent Cell Imaging. J. Biomed. Nanotechnol..

[85] Yang G., Shi J., Wang S., Xiong W., Jiang L., Burdab C., Zhu J. (2013). Fabrication of a boron nitride–gold nanocluster composite and its versatile application for immunoassays. Chem. Commun..

[86] Gomez J.L. (2013). Zinc Oxide Nanostructures: From Growth to Application. J. Mater. Sci..

[87] Yuan Y., Wu S., Shu F., Liu Z. (2014). An MnO_2_ Nanosheet as a Label-Free Nanoplatform for Homogeneous Biosensing. Chem. Commun..

[88] Zhai W., Wang C., Yu P., Wang Y., Mao L. (2014). Single-Layer MnO_2_ Nanosheets Suppressed Fluorescence of 7-Hydroxycoumarin: Mechanistic Study and Application for Sensitive Sensing of Ascorbic Acid *in Vivo*. Anal. Chem..

[89] He D., He X., Wang K., Yang X., Yang X., Li X., Zou Z. (2014). Nanometer-Sized Manganese Oxide-Quenched Fluorescent Oligonucleotides: An Effective Sensing Platform for Probing Biomolecular Interactions. Chem. Commun..

[90] Balendhran S., Walia S., Alsaif M., Nguyen E.P., Ou J.Z., Zhuiykov S., Sriram S., Bhaskaran M., Kalantarzadeh K. (2013). Field Effect Biosensing Platform Basedon 2D α-MoO_3_. Acsnano.

[91] Yakimova R., Selegard L., Khranovskyy V., Pearce R., Spetz A.L., Uvdal K. (2012). ZnO materials and surface tailoring for biosensing. Front. Biosci..

[92] Vabbina P.K., Kaushik A., Pokhrel N., Bhansali S., Pala N. (2015). Electrochemical Cortisol Immunosensors Based on Sonochemically Synthesized Zinc Oxide 1D Nanorods and 2D Nanoflakes. Biosens. Bioelectron..

[93] Sticker D., Rothbauer M., Charwat V., Steinkühler J., Bethge O., Bertagnolli E., Wanzenboeck H., Ertl P. (2015). Zirconium Dioxide Nanolayer Passivated Impedimetric Sensors for Cell-Based Assays. Sens. Actuators B.

[94] Zhao Y., Zhao J., Li Y., Ma D., Hou S., Li L., Hao X., Wang Z. (2011). Room temperature synthesis of 2D CuO nanoleaves in aqueous solution. Nanotechnology.

[95] Sun S., Sun Y., Chen A., Zhang X., Yang Z. (2015). Nanoporous copper oxide ribbon assembly of free-standing nanoneedles as biosensors for glucose. Analyst.

[96] Bhattacharjee A., Ahmaruzzaman M. (2015). Facile synthesis of 2-dimensional CuO nanoleaves and their degradation behavior for Eosin Y. Mater. Lett..

[97] Bhattacharjee A., Ahmaruzzaman M. (2015). Green Synthesis of 2D CuO nanoleaves (NLs) and its application for the reduction of pnitrophenol. Mater. Lett..

